# Addressing the elephant in the room: Proceedings of the third annual Dengue Endgame Summit

**DOI:** 10.1371/journal.pntd.0014170

**Published:** 2026-04-01

**Authors:** Lauren E. Bahr, Alan L. Rothman, Alan D. T. Barrett, Darunee Buddhari, Derek A. T. Cummings, Rebecca C. Christofferson, Albert I. Ko, Eng Eong Ooi, Kathryn B. Anderson, Stephen J. Thomas, Adam T. Waickman

**Affiliations:** 1 Department of Microbiology and Immunology, State University of New York Upstate Medical University, Syracuse, New York, United States of America; 2 Department of Cell and Molecular Biology, Institute for Immunology and Informatics, University of Rhode Island, Kingston, Rhode Island, United States of America; 3 Sealy Institute for Vaccine Sciences and Department of Pathology, University of Texas Medical Branch, Galveston, Texas, United States of America; 4 Department of Virology, Armed Forces Research Institute of Medical Sciences, Bangkok, Thailand; 5 Johns Hopkins Bloomberg School of Public Health, Baltimore, Maryland, United States of America; 6 Department of Pathobiological Sciences, Louisiana School of Veterinary Medicine, Baton Rouge, Louisiana, United States of America; 7 Department of Epidemiology of Microbial Diseases, Yale School of Public Health, New Haven, Connecticut, United States of America; 8 Instituto Gonçalo Moniz, Fundação Oswaldo Cruz, Salvador, Brazil; 9 Program in Emerging Infectious Diseases, Duke-NUS Medical School, Singapore, Singapore; 10 Global Health Institute, State University of New York Upstate Medical University, Syracuse, New York, United States of America; University of Central Florida, UNITED STATES OF AMERICA

## Abstract

Dengue is a disease caused by four serologically and genetically distinct orthoflaviviruses spread by mosquitoes. It is the largest arthropod-borne viral disease in the world with millions of human cases each year. The third annual Dengue Endgame Summit was convened in Syracuse, New York, over August 12–14, 2025, to discuss the current state of dengue, global dengue control efforts, and paths towards achieving sustained and effective dengue control. The event brought together 181 participants from 48 institutions across 11 countries to address current dengue outbreaks, next-generation mosquito vector interventions, vaccine and antiviral development, immune correlates of protection, and human challenge models. Four key themes emerged from the in-depth and nuanced discussions at the summit: (1) dengue control will require globally coordinated but locally tailored approaches that account for regional transmission differences and diverse circulating viral serotypes and genotypes; (2) the evolving dengue burden necessitates the strategic deployment of countermeasures, including vaccines, antivirals, and vector control, matched to local epidemiology; (3) longstanding dogma surrounding dengue immunopathogenesis and immune correlates of protection should be systematically reevaluated in light of advances in our understanding of cellular immunity, trained innate immunity, and the interplay of multiple components of the immune response; and (4) sustained progress depends on multisectoral collaboration, adequate financial resources, political commitment, and integration with broader vector-borne disease control efforts. These discussions highlighted both the complexity of dengue control and the diverse opportunities for accelerating progress toward a dengue endgame.

## Introduction

The third annual Dengue Endgame Summit was held in Syracuse, New York, from August 12th to 14th, 2025. Hosted by the SUNY Upstate Medical University’s Global Health Institute, the event brought together 181 attendees from 5 continents, 11 countries, and 48 institutions. During the event, participants evaluated the current state of dengue around the world, discussed progress toward achieving sustained dengue control, highlighted unique and evolving challenges facing the field, and explored persisting dogma across a number of topics in the dengue field. In-depth discussions covered recent large dengue virus (DENV) outbreaks in Brazil and Bangladesh, next-generation vector control efforts, ongoing vaccine and antiviral development efforts, growing understanding of immune correlates of protection, implications of data from dengue human challenge models, and late-breaking research from scientists across the world.

The summit opened with reflections on a dengue “endgame” from Alan Rothman of the University of Rhode Island. Dr. Rothman suggested—drawing from the analogy to a game of chess—the dengue field has progressed through the opening of its struggle against DENV and is currently in the middlegame, wherein the pieces are positioned on the board, but specific trajectories are undefined and the outcome is uncertain. He traced the history of dengue and how this collective experience has shaped the field’s current approach to countermeasure development and deployment efforts. He also addressed some of the challenges associated with achieving dengue control, using the parable of blind men identifying an elephant. As with the blind men encountering different parts of the elephant, disparate and superficially discordant observations in the dengue field may all be correct and valid in isolation, yet require a broader and unbiased perspective to accurately contextualize and bring the vision into focus. Dengue is a complex and growing global public health challenge, and to make tangible progress in prevention and treatment, Dr. Rothman emphasized that it will be necessary to integrate observations, analytical approaches, and intervention strategies from diverse perspectives and multiple disciplines.

To this end, over three days, summit attendees sought to better understand and describe the “elephant in the room”, discussing the field’s current challenges, tools for disease prevention and treatment, and evolving implementation strategies. Here, we highlight the four main takeaway messages from the third Annual Dengue Endgame Summit.

## Message #1: Dengue control will require globally coordinated but locally tailored approaches

The summit began with a reality check of the current dengue situation on the ground, highlighting both conserved public health features of dengue around the world as well as region-specific differences and challenges. Participants reviewed and discussed the challenges and opportunities in countries with both historically high levels of DENV circulation and those where DENV is a newly emerging infectious disease threat. Specifically, the state of DENV transmission in Brazil, Bangladesh, and Kenya was discussed, highlighting the differences in DENV transmission, risk, and impact across different geographical locations with differing levels of transmission and baseline immunity to DENV.

The recent explosive and record-breaking dengue outbreaks in Brazil were highlighted as an example of the challenge posed by the virus in a region with significant population-level immunity and rapidly evolving vector distribution. Brazil experienced its worst dengue season on record in 2024, with over 9.6 million cases and over 5,000 related deaths, a significant increase from the approximately 1.66 million suspected cases and roughly 1,000 deaths recorded in 2023 [1]. While the root causes for this outbreak are complex and hotly debated, climate conditions facilitating year-round conditions for transmission and the expansion of *Aedes aegypti* into previously unaffected southern temperate regions were cited as significant contributing factors. Despite these challenges, large-scale real-world vaccine deployment and novel vector control efforts are ongoing and show promise in limiting disease in the face of growing DENV transmission [2,3].

Although separated by nearly 10,000 miles, conceptual parallels were drawn between the dengue situations in Brazil and Bangladesh, with several exceptions highlighting unique regional challenges posed by the disease. Bangladesh exemplifies the shifting face of DENV epidemiology in a country where the climate is favorable for *Aedes* mosquitoes 9 months of the year. While seasonal transmission of DENV has been observed in Bangladesh since the 1960s, the burden of dengue has increased dramatically since 2022, resulting in an estimated 2.4 million annual infections and over 2,000 deaths between 2000 and 2025 [4]. While the etiology of this increase in DENV transmission is complex, increasing regional temperatures and changes in rainfall patterns have been linked to longer DENV transmission seasons and the emergence of DENV in regions of the country previously not impacted. In addition, the emergent co-circulation of multiple DENV serotypes in the region has increased the probability of heterotypic secondary infections [5]. In contrast, sustained low-level DENV transmission has been observed for decades in coastal Kenya, but to date dengue has not yet represented a significant public health burden despite apparent sustained endemic transmission [6,7]. However, the recent explosive outbreak of Chikungunya virus (CHIKV), another arbovirus transmitted by *Aedes aegypti*—as well as the persistent burden posed by malaria—emphasizes that the conditions in Kenya and other parts of Eastern Africa are ripe for increased DENV transmission, and vigilance is called for to ensure that outbreaks are detected and addressed.

While geographical differences were identified and specific regional needs were highlighted, the importance of global coordination was also apparent. Demographic differences, differences in circulating DENV serotypes/genotypes, differences in reporting strategies/requirements, as well as differences in population-level immunity have significant implications for where and how DENV countermeasures can be applied for maximal effect. Defining severe disease, disseminating information on detecting the disease, understanding circulating serotypes and strains for accurate countermeasure development, and providing the necessary resources to combat DENV transmission will require global communication and coordination.

## Message #2: The evolving and changing dengue burden necessitates strategic deployment of countermeasures

The burden of dengue has evolved as demographics, population-level immunity, and force of infection have changed around the world [8,9]. What was once considered a pediatric infectious disease threat is now extending its impact to an aging population, with the burden of disease in some regions shifting to older individuals with complex co-morbidities and immunologic backgrounds [10,11]. This demographic shift—which is occurring at different rates in different regions of the world—has significant implications for when, where, and how countermeasure deployment efforts can have the most significant public health impact.

Notably, it is recognized that suppressing the force of DENV infection alone can lead to both a shift in infection demographics towards an older demographic and an increase in the frequency of severe dengue in older adults. A prime example of this phenomenon has been observed in Singapore, where stringent vector control practices have resulted in extremely low *Aedes* density [12]. These measures profoundly decreased DENV exposure in the population, greatly reducing the burden of disease experienced over the last few decades. However, the prevention of infections earlier in life has led to symptomatic primary and post-primary DENV infections occurring in an aging population who are at inherently higher risk of developing more severe disease due to higher prevalence of comorbidities [12]. This highlights the need for strategic countermeasure deployment, continued epidemiological assessment of populations at risk, and emphasizes the importance of synergistic interventions.

Factors such as a changing climate and resource equity must also be considered when designing and deploying countermeasures. For example, the controlled release of wMel *Wolbachia*-infected *Aedes* mosquitoes—in which wMel-infected females are highly resistant to DENV infection and matings between wMel-infected males and uninfected females produce non-viable eggs—is being assessed in multiple regions around the world [13,14]. However, the magnitude and durability of introgression appear to be variable and dependent on climate and microclimate features (as the stability of wMel introgression is temperature dependent), as well as access to roads where vehicles could safely traverse to dispense infected mosquitoes. Furthermore, how wMel-mediated vector suppression—wherein the release of wMel *Wolbachia*-infected male *Aedes* mosquitoes is used to suppress vector reproduction without the need for stable introgression—must be considered in light of recent successes in Singapore [15]. Equitable access to countermeasures must be considered during development and deployment, and a mix of vector control and vaccines may provide the greatest likelihood of mitigating the dengue burden.

## Message #3: Historic understandings of dengue transmission, immunopathogenesis, and disease require systematic re-evaluation

Longstanding dogma surrounding DENV transmission, pathogenesis, immune correlates, and serology has historically guided the study and diagnosis of dengue, as well as countermeasure development efforts. These insights, while informative and representative of state-of-the-art understandings at the time of their conception, need to be reevaluated in light of new technology, data, and an ever-changing global dengue landscape in which the four DENVs now circulate. Notably, attendees highlighted the risk of over-reliance on reductionist models of immune-mediated enhancement of dengue severity. While immune correlates of risk and/or protection from DENV infection remain incompletely understood, recent advances in our understanding of cellular immunity [16–18], trained innate immunity [19], and multiparametric serology [20,21] offer hope that a more holistic understanding of DENV immunity and immunopathogenesis may be achievable. As such, there was also a call for the field to rely more on descriptive language when making associations between clinical outcomes and immunologic endpoints versus reflexing to commonly used terms which may or may not apply (i.e., immune enhancement).

Summit attendees largely agreed that a single immune correlate of protection from infection is unlikely to be identified or relevant considering the immunologic complexity of dengue and the contribution and co-circulation of multiple DENV serotypes and genotypes. Furthermore, reliance on neutralizing antibody titers as a sole readout of DENV immunity and prior DENV exposure—especially in the setting of vaccine evaluation—must be systematically reevaluated; in particular, as either a correlate or surrogate of protection. Advances in human challenge models offer hope for understanding the biology of DENV infection and disease pathogenesis but need to be paired with robust epidemiologic studies to truly understand individual- and population-level correlates of risk and immunity.

## Message #4: The dengue field requires advocacy, aggressive strategic communications, international coordination, and leadership

Given the many complexities in combating DENV transmission raised during the discussions, summit attendees addressed the broader question—what does a “dengue endgame” look like? What is needed to get there? A main takeaway from this year’s summit was that attendees believe that strategic communication and advocacy are needed in order to make meaningful and sustained progress. With a changing climate that is broadening the geographic range of vectors capable of transmitting DENVs and the continuing increase in the annual number of DENV cases, communications globally and locally will play a large role in dengue prevention. Goals for prevention and disease metrics need to be defined, resources and ideas need to be shared, and treatment and information must be made accessible to the public on a global level. Experts in the field have called for interventions that will prevent severe disease requiring hospitalization, which is challenging as the very definition of severe dengue continues to be debated. Global networks have been formed with the goal of creating shared clinical definitions, platforms for equitable access and distribution of medical countermeasures, and resource sharing. Initiatives such as the Pan American Dengue Research Network and the Asia Dengue Summit [22–24] have also provided crucial platforms for regional coordination. Integration of findings and strategies across these efforts can amplify global impact and ensure diverse regional perspectives are represented in endgame strategies. This work is ongoing but has been impactful thus far, with global alliances involved in countermeasure efficacy trials. Concomitant with this theme, strategic global coordination of scientific, public health, and science support functions is needed in order to meet the aggressive goals outlined above.

## Conclusions

As dengue cases continue to rise and transmission expands into new regions of the world, scientists, clinicians, modelers, vector control experts, and epidemiologists are working together to decrease transmission and the resulting disease. The annual Dengue Endgame Summit offered an opportunity to come together and reflect on successes, challenges, and goals for the field’s future.

Historically, the dengue field has approached control and countermeasures development efforts in a siloed, region-specific manner, without large-scale consensus or global communication. Critically, this is changing. New consortia and collaborative efforts are growing worldwide. These connections must continue to strengthen if we are to see significant impacts on severe dengue cases and transmission. Of paramount importance, the field needs to work together globally to share and ensure coordinated, effective, and accessible countermeasure development and testing, while still remaining responsive and cognizant of regional-specific needs and requirements. Classic dogma should be challenged as new data and models are developed and revised when necessary. While many challenges remain, there was enthusiasm by attendees to the summit that the transition from middlegame to endgame is underway and—unlike in chess—the clock cannot be paused. The field must now convert strategic positioning into decisive action.

## References

[1] Sansone NMS, Boschiero MN, Marson FAL. Dengue outbreaks in Brazil and Latin America: the new and continuing challenges. Int J Infect Dis. 2024;147:107192. doi: 10.1016/j.ijid.2024.107192 39067668

[2] Ranzani OT, Lazar Neto F, Mareto LK, Brumatti TS, de Oliveira RD, da Silva PV, et al. Effectiveness of the TAK-003 dengue vaccine in adolescents during the 2024 outbreak in São Paulo, Brazil: a test-negative, case-control study. Lancet Infect Dis. 2026;26(1):91–100. doi: 10.1016/S1473-3099(25)00382-2 40845862

[3] de Morais Batista F, Carcamo PM, Nelson E, da Silva Neto AB, Tsuha DH, Lahdo V, et al. The impact of large-scale release of Wolbachia mosquitoes on dengue incidence in Campo Grande, Brazil: an ecological study. Lancet Reg Health Am. 2025;54:101327. doi: 10.1016/j.lana.2025.101327 41438239 PMC12719740

[4] Salje H, Paul KK, Paul R, Rodriguez-Barraquer I, Rahman Z, Alam MS, et al. Nationally-representative serostudy of dengue in Bangladesh allows generalizable disease burden estimates. Elife. 2019;8:e42869. doi: 10.7554/eLife.42869 30958263 PMC6513551

[5] Ogieuhi IJ, Ahmed MM, Jamil S, Okesanya OJ, Ukoaka BM, Eshun G, et al. Dengue fever in Bangladesh: rising trends, contributing factors, and public health implications. Trop Dis Travel Med Vaccines. 2025;11(1):26. doi: 10.1186/s40794-025-00251-6 40784902 PMC12337422

[6] Muthanje EM, Kimita G, Nyataya J, Njue W, Mulili C, Mugweru J, et al. March 2019 dengue fever outbreak at the Kenyan south coast involving dengue virus serotype 3, genotypes III and V. PLOS Glob Public Health. 2022;2(3):e0000122. doi: 10.1371/journal.pgph.0000122 36962260 PMC10021577

[7] Blaylock JM, Maranich A, Bauer K, Nyakoe N, Waitumbi J, Martinez LJ, et al. The seroprevalence and seroincidence of dengue virus infection in western Kenya. Travel Med Infect Dis. 2011;9(5):246–8. doi: 10.1016/j.tmaid.2011.06.005 21778117

[8] Vicco A, McCormack C, Pedrique B, Ribeiro I, Malavige GN, Dorigatti I. A scoping literature review of global dengue age-stratified seroprevalence data: estimating dengue force of infection in endemic countries. EBioMedicine. 2024;104:105134. doi: 10.1016/j.ebiom.2024.105134 38718682 PMC11096825

[9] Huang AT, Buddhari D, Kaewhiran S, Iamsirithaworn S, Khampaen D, Farmer A, et al. Reconciling heterogeneous dengue virus infection risk estimates from different study designs. Proc Natl Acad Sci U S A. 2025;122(1):e2411768121. doi: 10.1073/pnas.2411768121 39739790 PMC11725863

[10] Ng WY, Atan R, Mohd Yunos N, Bin Md Kamal AH, Roslan MH, Quah KY, et al. A double whammy: the association between comorbidities and severe dengue among adult patients-A matched case-control study. PLoS One. 2022;17(9):e0273071. doi: 10.1371/journal.pone.0273071 36126060 PMC9488767

[11] Huang AT, Takahashi S, Salje H, Wang L, Garcia-Carreras B, Anderson K, et al. Assessing the role of multiple mechanisms increasing the age of dengue cases in Thailand. Proc Natl Acad Sci U S A. 2022;119(20):e2115790119. doi: 10.1073/pnas.2115790119 35533273 PMC9171776

[12] Ting R, Dickens BL, Hanley R, Cook AR, Ismail E. The epidemiologic and economic burden of dengue in Singapore: a systematic review. PLoS Negl Trop Dis. 2024;18(6):e0012240. doi: 10.1371/journal.pntd.0012240 38857260 PMC11192419

[13] Collins MH, Potter GE, Hitchings MDT, Butler E, Wiles M, Kennedy JK, et al. EVITA Dengue: a cluster-randomized controlled trial to EValuate the efficacy of Wolbachia-InfecTed *Aedes aegypti* mosquitoes in reducing the incidence of Arboviral infection in Brazil. Trials. 2022;23(1):185. doi: 10.1186/s13063-022-05997-4 35236394 PMC8889395

[14] Utarini A, Indriani C, Ahmad RA, Tantowijoyo W, Arguni E, Ansari MR, et al. Efficacy of wolbachia-infected mosquito deployments for the control of dengue. N Engl J Med. 2021;384(23):2177–86. doi: 10.1056/NEJMoa2030243 34107180 PMC8103655

[15] Lim JT, Chong CS, Chang CC, Mailepessov D, Dickens B, Lai YL, et al. Dengue suppression by male wolbachia-infected mosquitoes. N Engl J Med. 2026. doi: 10.1056/NEJMoa2503304 41671481

[16] Gregorova M, Santopaolo M, Garner LC, Hayati RF, Diamond D, Ramamurthy N, et al. Early NK-cell and T-cell dysfunction marks progression to severe dengue in patients with obesity and healthy weight. Nat Commun. 2025;16(1):5569. doi: 10.1038/s41467-025-60941-9 40595657 PMC12214611

[17] Gonnella G, Libri V, Gioacchino E, Mella S, Sann S, Sorn S, et al. Immune profiling in subclinical secondary dengue-infected cases reveals adaptive immune signatures correlated to protection from severe dengue. Cell Host Microbe. 2025;33(7):1191-1207.e4. doi: 10.1016/j.chom.2025.06.006 40580952 PMC12757132

[18] Ghita L, Yao Z, Xie Y, Duran V, Cagirici HB, Samir J, et al. Global and cell type-specific immunological hallmarks of severe dengue progression identified via a systems immunology approach. Nat Immunol. 2023;24(12):2150–63. doi: 10.1038/s41590-023-01654-3 37872316 PMC10863980

[19] Ong EZ, Yee JX, Koh CW, Ooi JS, Tham CYL, Chan KR, et al. Dengue virus infection reprograms baseline innate immune gene expression. Med. 2025;6(11):100841. doi: 10.1016/j.medj.2025.100841 40925380

[20] Dias AG Jr, Atyeo C, Loos C, Montoya M, Roy V, Bos S, et al. Antibody Fc characteristics and effector functions correlate with protection from symptomatic dengue virus type 3 infection. Sci Transl Med. 2022;14(651):eabm3151. doi: 10.1126/scitranslmed.abm3151 35767652 PMC10115655

[21] Dias AG Jr, Duarte EM, Zambrana JV, Cardona-Ospina JA, Bos S, Roy V, et al. Anti-dengue virus antibodies that elicit complement-mediated lysis of Zika virion correlate with protection from severe dengue disease. Cell Rep. 2025;44(5):115613. doi: 10.1016/j.celrep.2025.115613 40333188

[22] Srisawat N, Gubler DJ, Pangestu T, Thisyakorn U, Ismail Z, Goh D, et al. Trop Med Infect Dis. 2023;8(4). doi: 10.3390/tropicalmed8040231 37104356 PMC10142460

[23] Srisawat N, Gubler DJ, Pangestu T, Limothai U, Thisyakorn U, Ismail Z, et al. Proceedings of the 6th Asia Dengue Summit, June 2023. PLoS Negl Trop Dis. 2024;18(3):e0012060. doi: 10.1371/journal.pntd.0012060 38551892 PMC10980189

[24] Ismail Z, Gubler DJ, Pangestu T, Thisyakorn U, Srisawat N, Goh D, et al. Proceedings of the 7(th) Asia Dengue Summit, June 2024. Vaccines (Basel). 2025;13(5). doi: 10.3390/vaccines13050493 PMC12115888 40432105

